# Multiorgan immunohistochemical endothelial expression of E-selectin in a forensic case of sepsis

**DOI:** 10.1007/s12024-023-00663-w

**Published:** 2023-06-08

**Authors:** Simone Bohnert, Stefanie Trella, Ulrich Preiß, Michael Bohnert, Michael Tsokos, Helmut Heinsen

**Affiliations:** 1 Institute of Forensic Medicine, University of Wuerzburg, Versbacher Str. 3, Wuerzburg, 97078 Germany; 2 Institute of Legal Medicine and Forensic Sciences, Charité – Universitätsmedizin Berlin, Turmstr. 21 (Haus N), Berlin, 10559 Germany

**Keywords:** Sepsis, E-selectin, Forensic neuropathology, Cerebrospinal fluid

## Abstract

Sepsis is one of the major threats for the survival and prognosis of patients in intensive care units. In cases where detailed clinical data and monitoring is available, the diagnosis of sepsis is reliable. But when clinical data are incomplete or missing and sepsis is only suspected based on the autopsy results, the picture is often equivocal. This report describes the gross pathological findings obtained from the autopsy of a 48-year-old woman with Crohn’s disease after surgical intervention. Macroscopically, we found intestinal perforation and signs of peritonitis. Histologically, the pulmonary/bronchial arteries were lined with E-selectin (CD 62E)-positive endothelial cells, which are an established postmortem histological marker of sepsis. We extended our investigations to the cerebral cortex and subcortical medullary layer. The endothelium of the cortical vessels and those in the cerebral medullary layer were likewise immunopositive for E-selectin. Furthermore, numerous TMEM119-positive, highly ramified microglial cell profiles were found in the grey and white matter. Microglial cells were lining the vascular profiles. In addition, TMEM119-positive microglial profiles were abundant in the cerebrospinal fluid (CSF). Multiorgan E-selectin positivity of the vascular endothelia provides further evidence for the postmortem diagnosis of sepsis.

## Case report

### Macroscopic findings

This report deals with the death of a 48-year-old woman with Crohn’s disease in a maximum care hospital. The clinical diagnosis was intestinal perforation, peritonitis, sepsis, and death by multiorgan failure after surgical intervention.

**Fig. 1:**
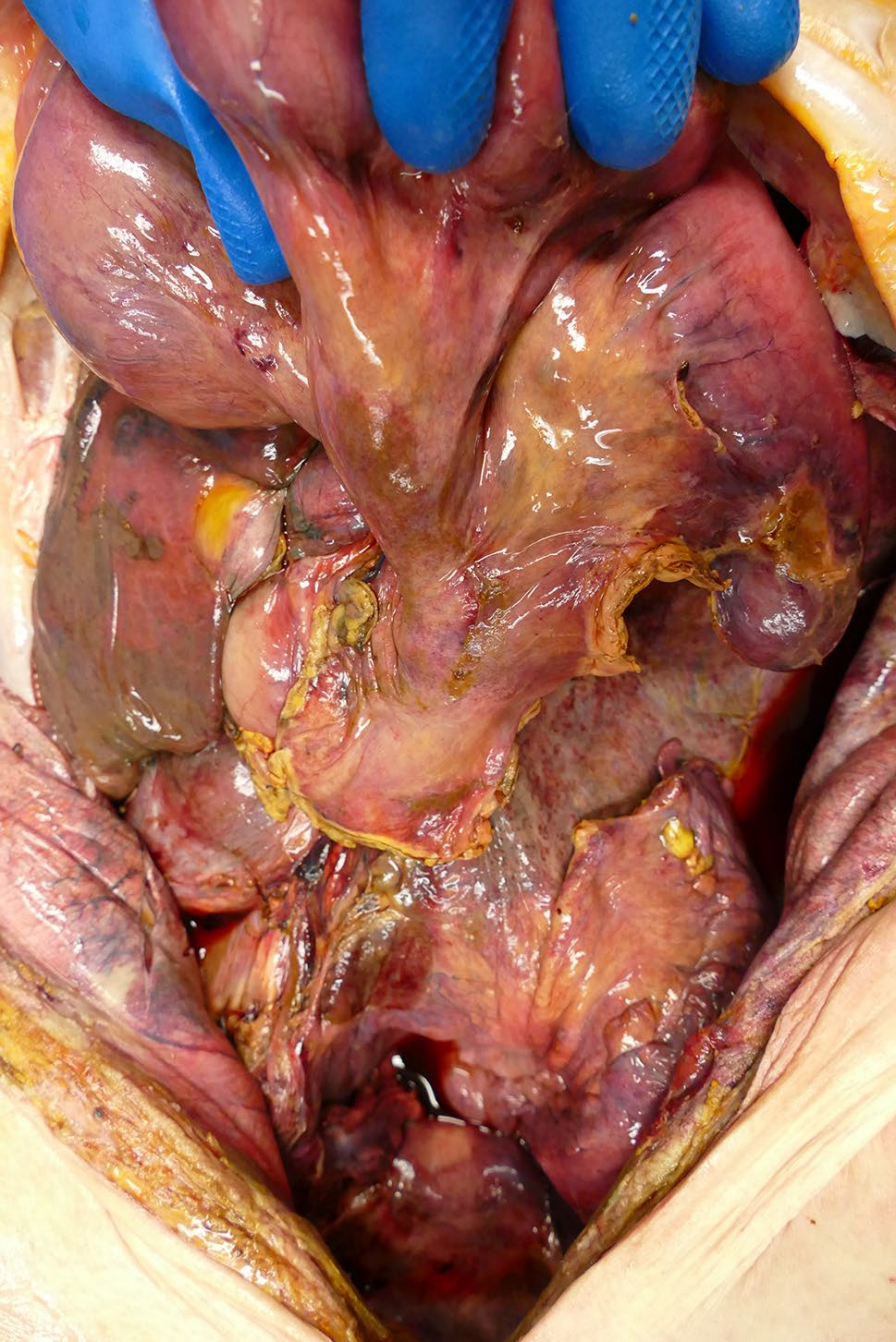
General reddening of the remaining intestine and peritoneum with a piebald yellowish to grayish coating

Three days postmortem, an autopsy was performed on the deceased, who was 168 cm tall and had a weight of 48 kg. On external inspection, the deceased was found to be in a severely reduced general and nutritional condition. There was a 27-cm-long and 13-cm-wide gaping surgical cut running mediosagittaly from the pubic symphysis to the xiphoid process of the sternum. The depth of the wound was covered by smeary, partially watery tissue. Inspection of the abdominal cavity showed that large portions of the small and large intestine except the S-shaped curved rectal segment had been surgically removed. Smooth, inconspicuous deposition margins of the intestinal suspension existed. The deposition of the small bowel was blindly closed with sufficient suture. The remaining intestine and peritoneum was generally reddened with a piebald yellowish to grayish coating (Fig. 1). The liver (2350 g) and spleen (210 g) were considerably enlarged. The cut surface of the former had a mottled appearance. The latter was characterized by a firm consistency. The white pulp could not be identified after dissection. Both thoracic cavities contained about 400 ml of an amber-colored watery fluid with mucilaginous filaments of yellowish color. The lungs (left: 800 g; right: 940 g) had a water-pad-like consistency and showed numerous punctate hemorrhages of the pleura and air-filled bubbles arranged in a bead-like manner at the edges of the lobes. The mucous membranes of major parts of the trachea and the bronchi appeared hyperemic, and the trachea, the bronchial tree, and the alveoli were filled with a mucinous reddish fluid.

### Microscopic findings and additional clinical biochemical analysis

**Fig. 2:**
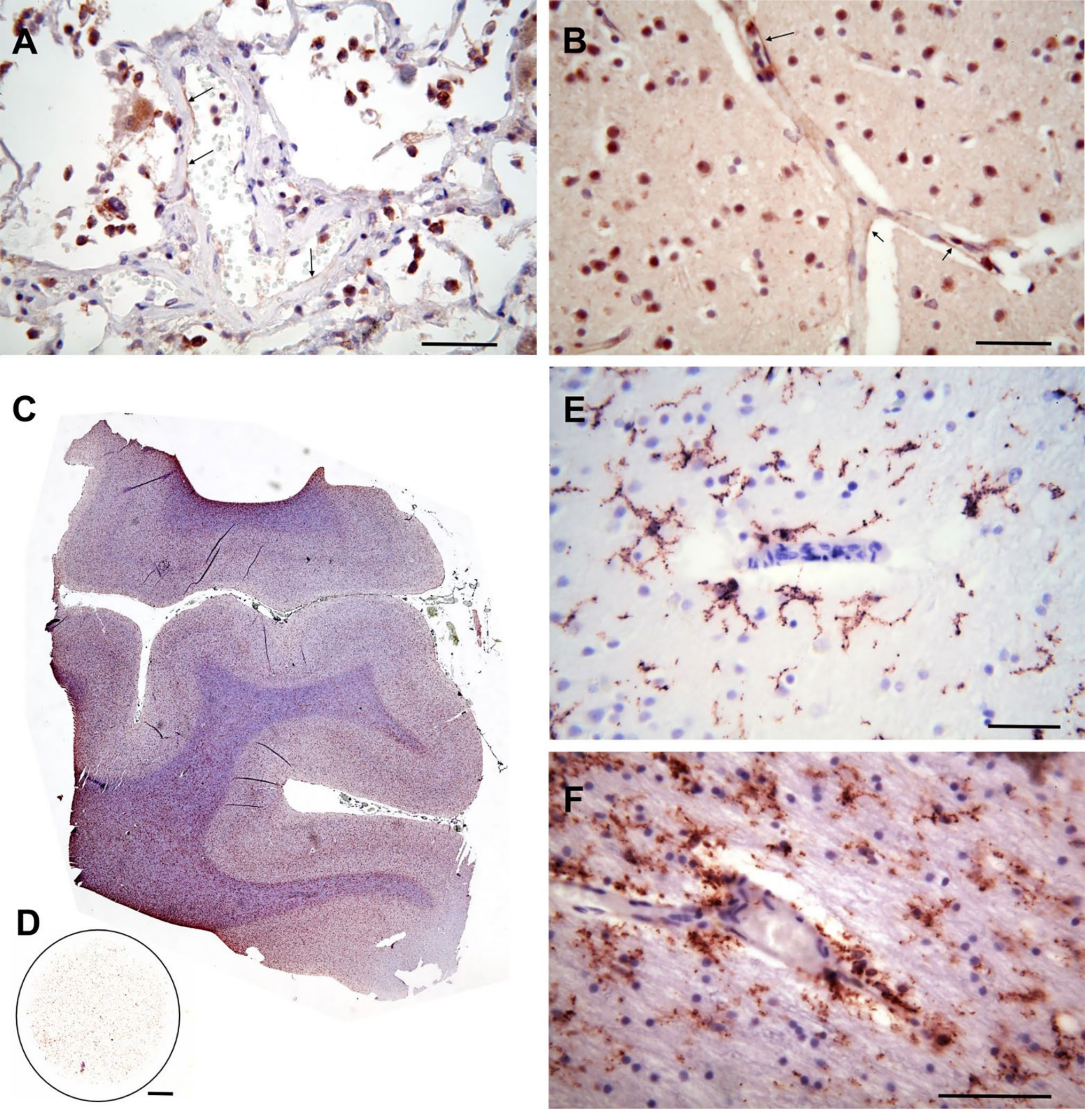
**A** Lung, hematoxylin-eosin (H.E), E-selectin (CD 62E) immunohistochemistry (IH). Small arrows point to E-selectin-positive endothelial cytoplasm. **B** Cerebral cortex, dorsomedial parietal lobe, H.E., E-selectin IH. Arrows point to E-selectin-positive endothelial cytoplasm. **C** Gyri from the dorsomedial parietal lobe, TMEM119 IH, H.E. **D** TMEM119 immunocytochemistry in cytospin preparation from the CSF obtained during autopsy. **E** High-power magnification from Fig. C).TMEM119 IH, H.E. Cortical microglia. **F** Same region and staining as in **C**. Microglia from the medullary layer. Bar in **A**, **B**, **E,** and **F** = 50 μm, in **C** and **D** = 1 mm

Small routine diagnostic tissue samples of the brain (dorsomedial frontal and parieto-occipital lobes) and of all lobes of the lung were formalin fixed, embedded in paraffin, cut into 5 μm-thick slices, and stained with hematoxylin-eosin and immunohistochemically with E-selectin (CD 62E) and TMEM119. Clusters of granulocytes with varying cellular density were encountered in all parts of the bronchial tree including the alveoli, which were frequently crammed with numerous leucocytes and intermingled alveolar phagocytes. After immunohistochemical staining with an antibody against E-selectin, the delicate endothelium of small bronchial/pulmonary arteries stained positively (Fig. 2A arrows). The same was true for cortical and subcortical small vessels (Fig. 2B).

At low magnification, the cortex and the subcortical medullary layer were characterized by a high density of TMEM119-positive microglial cell profiles (Fig. 2C). A large number of freely floating microglial cell profiles was also present in the cytospin preparation of the cerebrospinal fluid (CSF; Fig. 2D). Microglial cells appeared to be arranged in a laminar pattern in the border regions above and below layer IV in this transverse section through the dorsomedial parietal cortex. In addition, microglial cells were associated with cortical and subcortical vessels (Fig. 1E, F).

The procalcitonin (PCT) concentration in blood samples taken from the femoral vein of the deceased was 0.92 ng/ml.

## Discussion

Sepsis is a major threat for the survival and prognosis of patients in intensive care units. According to the new definition, sepsis is a life-threatening organ dysfunction caused by an aberrant immune response to infection that is responsible for the pathogenesis and progression of sepsis [1, 2]. The interplay between malfunctions in the immune system and multiple organ damage is deemed the major cause of the poor outcome of sepsis cases. In postmortem cases with comprehensive clinical data, routine pathological examination will verify the clinical diagnosis. In forensic cases, however, the postmortem diagnosis of sepsis is difficult when there is no information on the preceding clinical course. Gross macroscopic findings and routine histology in cases of suspected fatal sepsis are most often unspecific and equivocal. Additional parameters are needed to verify a postmortem diagnosis of sepsis as the cause of death. The lungs prove to be particularly affected in the course of sepsis and the progressively impaired lung function is the major complication of the latter [3] Therefore, attention has been focused on the immunohistochemical detection of different markers of inflammatory cellular responses to sepsis in the lungs. E-selectin is a quiescent endothelial adhesion molecule. It can be immunohistochemically demonstrated after endothelial contact with cytokines and bacterial lipopolysaccharides [4]. Studies by Huang et al. [5] and Tsokos et al. [4] showed that endothelial activation and the expression of E-selectin correlate with an ongoing presence of cytokines.

In the present case, we found E-selectin-positive endothelial cells in both pulmonary and intracerebral vessels of a woman suffering from Crohn’s disease with complications of intestinal perforation and peritonitis. In addition, the expression of E-selectin was shown at the endothelium of cerebral vessels in a forensic context for the first time, suggesting neuropathological involvement of the central nervous system (CNS) in sepsis. Future studies should verify this novel observation in a case-control study.

The presence of E-selectin-positive endothelial cells in cerebral vessels was obviously chronologically and topographically paralleled by the enhanced density of TMEM119-positive microglial cell profiles in the brain and CSF. Microglia, the resident macrophages of the CNS, are considered the first cellular defense line whenever damage occurs, be it traumatic or inflammatory [6]. In 2016, TMEM119, a trans-membranous molecule, proved to be a specific and robust microglial marker [7]. Since that time, it has served for the immunohistochemical demonstration of microglial response to fatal traumatic brain injury (TBI) or intoxication [8, 9]. In addition, the assessment of the density of TMEM119-positive microglial cell profiles in cytospin preparations of the CSF after different fatalities and postmortem intervals (PMI) could be used as a marker for the evaluation of complex neuropathological processes in the CNS [10, 11].

The measured concentration of the biochemical sepsis marker PCT at 0.92 ng/ml is above the reference range (0–0.5ng). However, it is well below the values published by Tsokos of > 2 ng/ml or 100 ng/ml in severe cases [12]. These authors determined the concentration of PCT in the femoral blood immediately after autopsy, whereas our sample had been frozen for 48 h, thus exceeding the half-life of PCT, which is 25–30 h [12]. This difference between the reported values and the actual values could be caused by freezing and storing the sample.
